# Molecular Characterization of Lyssaviruses Originating from Domestic and Wild Cats Provides an Insight on the Diversity of Lyssaviruses and a Risk of Rabies Transmission to Other Susceptible Mammals and Humans in South Africa

**DOI:** 10.3390/pathogens12101212

**Published:** 2023-10-02

**Authors:** Kefentse Tsie, Ernest Ngoepe, Baby Phahladira, Nelisiwe Khumalo, Claude Sabeta

**Affiliations:** 1 WOAH Rabies Reference Laboratory, Agricultural Research Council (Onderstepoort Veterinary Research), Onderstepoort, Pretoria 0110, South Africa; 2 Central Veterinary Laboratory, Corner Althea Road and Central, Manzini H100, Eswatini; thandolwababe@gmail.com; 3 Department of Veterinary Tropical Diseases, University of Pretoria, Private Bag 04, Onderstepoort, Pretoria 0110, South Africa

**Keywords:** *Lyssavirus mokola*, domestic cat, African wildcat, rabies, Eswatini

## Abstract

Rabies is one of the most significant public and veterinary health problems, causing approximately 59,000 human deaths annually in the developing countries of Asia and Africa. The aetiologic agent, a viral species of the *Lyssavirus* genus, is highly neurotropic and has a wide host range, including terrestrial mammals and several *Chiropteran* species. The *Lyssavirus mokola* (MOKV) was first isolated in the late 1960s from organ pools of shrews (*Crocidura flavescens manni*) in the Mokola forest (Nigeria). To date, at least 30 MOKV isolations have been confirmed, all exclusively from Africa, with 73% from southern Africa. There is limited knowledge about the epidemiology of MOKV, and the reservoir host species is unknown. Here, we report on the molecular characterization of rabies viruses originating from both domestic and African wild cats. A partial region of the lyssavirus genome, encoding the nucleoprotein, was amplified and sequenced. Nucleotide sequence analysis demonstrated that 98% of cats were infected with both the canid and mongoose rabies virus variants, as well as a rare lyssavirus, *Lyssavirus mokola*, from a domestic cat from Eswatini. Furthermore, the nucleotide sequence divergence between the recently identified MOKV isolate and the historical *Lyssavirus mokola* isolates ranged from 6.8% to 8.3%. This study further highlights the association between the potential host species of *Lyssavirus mokola* and the domestic cat as an incidental host, and the important role cats may play in rabies transmission dynamics in the country. Therefore, continuous vaccination of domestic cats against rabies is crucial, even after the elimination of dog-mediated rabies, as spillover related to sylvatic rabies cycles is likely to occur.

## 1. Introduction

Rabies is a neglected zoonotic and fatal disease of all mammalian species including humans. The disease (rabies) remains the most significant public and veterinary health problem, and an estimated 59,000 humans succumb to the disease annually, particularly in the resource-limited developing countries of Asia and Africa [1]. The causative agent of the disease is a member of the *Lyssavirus* genus, currently composed of 17 confirmed viral species [2]. The prototype species is *Lyssavirus rabies*, and the rest are known as rabies-related viruses. Within the genus, there are two unclassified lyssaviruses, the Kotalahti bat lyssavirus (KBLV) isolated from a Brandt’s bat (*Myotis brandtii*) in Finland [3] and Matlo bat lyssavirus from a Natal long-fingered bat (*Miniopterus natalensis*) [4]; and both await ratification by the International Committee on Taxonomy of Viruses (ICTV). There are currently six documented *Lyssavirus* species identified and confirmed from terrestrial and chiropteran host species in Africa and these are the classical *Lyssavirus rabies*, rabies virus (RABV), *Lyssavirus lagos*, Lagos bat virus (LBV), *Lyssavirus mokola*, Mokola virus (MOKV), *Lyssavirus duvenhage*, Duvenhage virus (DUVV), *Lyssavirus shimoni*, Shimoni bat virus (SHIBV), and *Lyssavirus ikoma*, Ikoma virus (IKOV) (41). All known lyssaviruses have been isolated from bat species, except MOKV and IKOV, whilst the latter was a one-off isolation from an African civet (*Civettictis civetta*) in Tanzania [5,6]. 

Rabies virus is distributed globally with the exception of some nation islands such as New Zealand, Marshall Islands, and/or Papua New Guinea [7]. The aetiologic agent of this fatal zoonotic disease is a highly neurotropic virus that has a wide host range, including terrestrial mammals and several bat species [5]. Most mammals are susceptible hosts, and only some species within the *Carnivora* and *Chiroptera* orders are major rabies reservoir host species. For instance, in southern Africa, classical RABV occurs as two distinct variants maintained in *Carnivora* and *Herpestidae* families; and these are referred to as the canid and mongoose RABV biotypes, respectively [8,9,10]. The canid RABV biotype is maintained and transmitted primarily by domestic dogs and wildlife carnivore species such as black-backed jackal (*Canis mesomelas*), bat-eared fox (*Otocyon megalotis*), and more recently the aardwolf (*Proteles cristatus*). In contrast, the mongoose RABV biotype is maintained and transmitted by members of the *Herpestidae* family, especially the yellow mongoose (*Cynictis penicillata*). The mongoose rabies biotype comprises five distinct lineages associated with specific geographic areas in Zimbabwe (n = 1) and South Africa (n = 4) [9,11], with the dominant one overlying the highveld plateau of the Free State province in South Africa. Molecular clock analysis estimated the mongoose RABV variant to be approximately 200 years old, and this estimation aligns with the historical description of rabies in South African mongoose populations dating back to the early 1800s [11]. Apart from the maintenance reservoir host species, the canid and mongoose RABV biotypes infections are reported in several domestic and wildlife species, with some terrestrial mammals serving as dead-end hosts [12,13]. 

Mokola virus (MOKV) was first isolated in the late 1960s from organ pools of shrews (*Crocidura flavescens manni*) in the Mokola forest in Nigeria [14,15]. Thereafter, two additional cases were reported from two girls, a 3.5-year-old in 1968, and a 6-year-old in 1971, both from Nigeria [16,17]. In these MOKV human cases, the clinical symptoms were atypical for classical rabies virus infection [17]. These are the only known MOKV infections associated with humans in lyssavirus history. Further MOKV isolations were made from a shrew (*Crocidura sp*.) in 1978, Cameroon [18], from a rodent (*Lophuromys sikapusi*) in 1981, Central African Republic [19] and in Ethiopia in 1989 from a cat (*Feline catus*) [20]. In Zimbabwe, the MOKV isolations were made between 1981 and 1984 in the south-west regions of the country from six cats and one dog previously vaccinated against rabies [21,22], and later in 1993 from a domestic cat [23]. In South Africa, MOKV was first isolated in 1970 from a vaccinated domestic cat in KwaZulu Natal (KZN) province [24] but was only confirmed as MOKV using better diagnostic biologicals. Subsequent MOKV isolations were made from the KZN (n = 7), Eastern Cape (EC) (n = 5) as well as from the Mpumalanga (MP) (n = 2) Provinces [25,26,27,28,29]. The majority of MOKVs in South Africa were recovered from domestic cats, with the exception of a single case in a domestic dog from MP Province. To date, all MOKV infections have been reported from the African continent, which might suggest that this lyssavirus is unique to the continent. However, little is known about the epidemiology of this African lyssavirus despite that there are at least 30 confirmed MOKV isolations, of which majority of cases are originating from southern Africa. Furthermore, the problem of MOKV ecology is compounded by an unknown reservoir host species and limited surveillance throughout the African continent, as most lyssaviruses confirmed during rabies diagnostics are never further characterized into viral species. 

Globally, the occurrence of rabies in domestic cats (*Felis catus*) is less commonly reported than the domesticated dog [30]. Currently, there is no evidence to indicate that domestic cats and African wild cats (*Felis lybica*) are involved as natural reservoir host species of lyssaviruses in South Africa and only serve as dead-end or incidental hosts. The current rabies vaccines do not confer protection against MOKV infection as shown by incidental evidence of the fatal infections of domestic cats that were previously vaccinated against RABV with Rabisin [22,26,27,28,29]. It is plausible that the frequent contact between domestic cats and their respective owners may pose a potential public health risk. In this manuscript, we report on a retrospective molecular characterization of lyssaviruses recovered from domestic and African wild cats. This is the first study aimed to molecularly characterize rabies positive cases identified from domestic and African wild cats in order to understand the potential involvement of the feline species in the epidemiology of lyssaviruses in South Africa.

## 2. Materials and Methods

### 2.1. Viruses

A cohort of 51 lyssaviruses obtained from domestic and African wild cats and initially confirmed to be lyssavirus positive between 2010 and 2020 were selected from the World Organization for Animal Health (WOAH) rabies reference laboratory repository of the Agricultural Research Council-Onderstepoort Veterinary Institute (ARC-OVI) (Pretoria, South Africa) [30]. The variety of suspected rabies cases (from both domestic and wildlife species) were submitted by state veterinarians to the diagnostic laboratory for rabies confirmation as part of the national rabies surveillance programme. The epidemiological information of the specimens is shown in Table S1. 

### 2.2. Total RNA Extraction, RT-PCR, and Sequencing

Total viral RNAs were extracted from the original brain-infected tissues using Tri-Reagent (Sigma-Aldrich, St Louis, MO, USA) according to the manufacturer’s instructions. The extracted RNA was stored at −80 °C until required for further analyses. A reverse transcription polymerase chain reaction (RT-PCR) was performed using the 001Lys (annealing position 1–14 based on the Pasteur Virus genome sequence) and 550B (annealing position 647-666) primers targeting the partial nucleoprotein (N) gene of the viral genome [31]. In brief, cDNA was synthesized as follows, 5 µg of total RNA was denatured at 70 °C and annealed to 20 pmol of the forward primer (001Lys) during a 5-min incubation The reaction mixture was immediately cooled on ice and the RNA was reverse transcribed at 50 °C for 60 min in a 20 μL-reaction mixture containing 200 U Superscript III reverse transcriptase (Invitrogen, Waltham, MA, USA), 1X First-strand buffer, 10 mM deoxynucleotide triphosphates (dNTPs) (Invitrogen, USA), and 40 U of RNase inhibitor (Promega, Madison, WI, USA). After cDNA synthesis, the reaction mixture was deactivated at 85 °C for 5 min. Thereafter, polymerase chain reactions (PCRs) were performed in a 50 μL-volume containing 1X PCR reaction buffer, 1.5 mM MgCl_2_, 250 μM of each dNTP, 5 μL of cDNA, 1.25 U of Takara Taq DNA polymerase (Takara, Shiga, Japan), and 40 pmol of each of the forward and reverse primers (Inqaba Biotech, Pretoria, South Africa) and amplified as previously described [31]. The PCR products were electrophoresed in 1% agarose gels and subsequently purified using the PCR purification kit (Qiagen, Hilden, Germany) according to the manufacturer’s protocol. The purified PCR amplicons were sequenced using both the 001Lys and 550B primers as in the PCR reactions with the BigDye® Terminator v3.1 sequencing reaction kit (Applied Biosystems, Waltham, MA, USA) on an ABI 3100 automated sequencer (Applied Biosystems, USA) at Inqaba Biotech (Hatfield, Pretoria, South Africa).

### 2.3. Phylogenetic Analysis

The 3′ and 5′ ends of the nucleotide sequences were trimmed and ambiguous bases removed by using molecular evolutionary genetics analysis version X (MEGA X) [32]. The consensus sequences were assembled and aligned using the Clustal*W* algorithm of MEGA X and the phylogenetic trees were reconstructed using the neighbor-joining (NJ) method [33]. The topology of the reconstructed phylogenetic tree was validated with a 1000 bootstrap replicates [34] and 70% was considered as the cut-off value supporting a phylogenetic grouping.

## 3. Results

**Figure 1:**
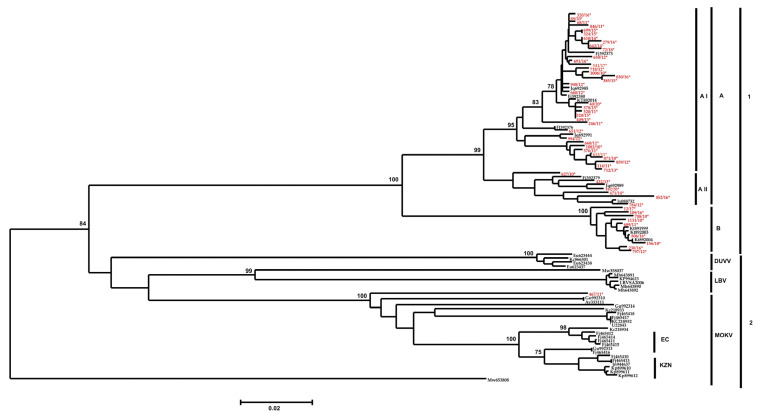
A neighbour joining phylogenetic tree of the partial N gene sequence of a common 540 bp region of the 51 rabies positive cases from feline species obtained from years 2010 and 2020 together with previously published lyssavirus sequences of the rabies virus (RABV), Mokola virus (MOKV), Duvenhage virus (DUVV) and Lagos bat virus (LBV). The viral isolates sequenced during the study a labelled in red with an asterisk symbol. A bootstrap value of 1000 was used to evaluate the branches and Matlo bat lyssavirus (MW653806) was used as an outgroup to root the tree. Note: DUVV denotes Duvenhage virus; LBV: Lagos bat virus; MOKV: Mokola virus; EC: Eastern Cape; KZN: KwaZulu Natal.

All the selected viruses were successfully amplified and yielded an expected amplicon of approximately 680 bp in size, and the sequences were trimmed to 540 bp for further analysis. The partial N-gene nucleotide sequences of the 51 samples were established and after the analyses, a lyssavirus sample (467/11) was confirmed to be a Mokola virus (MOKV) (Table S1). Given this new identification, all known and previously characterized and published sequence data of MOKV, Lagos bat virus (LBV) and Duvenhage virus (DUVV) isolates were included for additional and more comprehensive phylogenetic analyses (Table S2). The topology of the neighbour joining (NJ) and maximum likelihood (ML) phylogenetic trees were the same (ML tree not shown) and only the NJ phylogenetic tree is shown. The phylogenetic analysis revealed two distinct clusters 1 and 2, supported by a bootstrap value of 84% (Figure 1). The results further revealed that the viral isolates from cluster 1 belonged to rabies virus (RABV), whilst viral isolates in cluster 2 all belonged to rabies-related viruses included in the analysis (i.e., MOKV, LBV and DUVV isolates) (Figure 1). Cluster 1 could be further subdivided into two sub-clusters, shown as 1A and 1B, with a bootstrap value of 100% (Figure 1). It was found that the viral isolates in sub-cluster 1A belong to mongoose RABV variant, whereas viral isolates from sub-cluster 1B belong to the canid RABV variant. Sub-cluster 1A could be further delineated into two subgroups 1A (I and II) with bootstrap values of 99% (Figure 1). Sub-cluster 1A-I consists of viral isolates exclusively from the Free State and North-West, Mpumalanga and Northern Cape Provinces, whereas subcluster 1A-II consists of viral isolates (n = 9) from the Free State and Northern Cape Provinces respectively (Figure 1). Interestingly, viral isolates within the subgrouping 1A-I clustered according to their geographic locations of the Free State and North-West Provinces with a bootstrap of 78% respectively (Table S1). The data revealed that viral isolates in sub-cluster 1A were very closely related, with a 96% nucleotide sequence similarity, whereas sub-cluster 1B had 99% nucleotide sequence identity. In contrast, the data showed that viral isolates in sub-clusters 1A and 1B had 87% nucleotide sequence identity suggesting that the two clusters belong to distinct RABV variants (Figure 1). 

The phylogenetic data analysis showed that the rabies related viruses clustered together according to their viral species (e.g., DUVV isolates clustered together with a bootstrap support of 100%) (Figure 1). Further, phylogenetic analysis showed that the newly identified MOKV isolate (467/11) did not cluster with any of the previously identified MOKV lineages (Figure 1) but is part of a distinct and independent lineage. The phylogenetic data further demonstrated that MOKV isolates from South Africa were part of a compact geographic cluster, statistically supported by a bootstrap value of 100% (Figure 1). Furthermore, the South African MOKV isolates conformed to two different sub-clusters with a strong bootstrap support value of 100%, demonstrating a strong geographical determinant of the isolates from KwaZulu Natal (KZN) and Eastern Cape (EC) Provinces (Figure 1). The pairwise (P) distance comparisons between different MOKV isolates were performed on the highly conserved N gene region under study, and the pairwise matrix is shown (Table 1). The nucleotide sequence divergence values between the recently identified MOKV isolate from the Kingdom of Eswatini (467/11) and the historical Mokola viruses ranged from 6.8% to 8.3% (91.7% and 93.2% nucleotide sequence identity), with the highest divergence value of 8.3% observed between 467/11 (Eswatini) and 86101RCA (Central African Republic) (Table 1). The nucleotide sequence divergence between MOKV Eswatini and MOKV isolates from South Africa was 7.2% and 8.1% with Zimbabwean MOKV isolates on average (Table 1). Further, the nucleotide difference with Cameroon and Ethiopia isolates was 7.5% on average, respectively. It was apparent that the MOKV from the Kingdom of Eswatini was more closely related to the isolates from South Africa with a 93% sequence similarity on average than any other isolates (Table 1). 

## 4. Discussion

The primary objective of this study was to genetically characterize lyssaviruses confirmed from domestic and African wild cats with a view to understand the involvement of these species in lyssavirus epidemiology in South Africa. Our research clearly addressed the role of domestic and wild cats as potential transmitters of rabies viruses (RABVs) and/or non-rabies lyssaviruses to other mammals and humans across southern African country that is historically rich in lyssavirus diversity. This research complements the global elimination efforts of rabies in humans transmitted by dogs, since domestic cats have been demonstrated as effective rabies transmitters of major rabies aetiological agents prevalent across the world and notably in the Americas and Europe. In Zimbabwe, the annual incidence of cat rabies from 1950-1986 was 108, with 7of the cats confirmed to be infected with rabies-related viruses [22]. The cat’s ability to adopt a feral existence, and its close association with the smaller wildlife species on which it preys, as well as with man, gives it a unique place in the epidemiology of rabies. For instance, in Brazil, where the disease in dogs has been brought under control, the cat has emerged as an important reservoir host and potential vector of rabies to man [35]. The study further indicated that cat rabies cases between 2011 and 2022 were mostly caused by a lyssavirus variant commonly found in bats and accounted for 36/72 cases (50.0%). Thus, albeit cats not being true rabies reservoir hosts, they significantly play a role in rabies transmission to humans and other domestic species, potentially complicating the eradication of RABV in humans and other susceptible hosts species globally. 

Rabies in domestic cats is a potential public health concern given the association of this domestic carnivore with not only Mokola virus (MOKV) [36] but also with canid and mongoose RABV variants, which are zoonotic and important public health drivers in South Africa [37]. In comparison to a study performed in Brazil, results suggest that cats play a more important role in human rabies in Brazil than in South Africa. The reason for this difference may be that hematophagous (vampire) bats, which are considered to be important reservoirs and transmitters for the maintenance of sylvatic rabies in natural areas, have only been reported as inhabiting the Americas, with no occurrence in Africa. A previous study, though, demonstrated that despite close contact of humans with domestic cats, less than 3% of human rabies cases in the 36-year period were associated with exposure to these animals [37]. Interestingly, findings from our study showed that the majority of the cats were infected with rabies virus (RABV), i.e., both canid and mongoose RABV variants, as well as a single case of MOKV isolate. Our findings seem to suggest that domestic and wild cats interact with maintenance hosts of both the canid and mongoose rabies variants in nature. Surprisingly, the RABV isolates included in the phylogenetic analysis indicated that the majority of rabies cases in cats were associated with the mongoose RABV variant rather than the canid RABV variant. This could be attributed to several factors suggesting that some of the rabies cases occurred mostly in the farming communities across the country where the mongoose RABV variant is predominant or common. The discovery of different major RABV clades in felines analyzed here is of significant public and animal health importance given the undeniable record of a higher number of cases (in order of thousands in mammals and humans in Africa alone), compared to the negligible number of cases in bats and other mammals (including humans) caused by non-rabies lyssaviruses globally.

The domestic cats could have been in contact with the natural host species of MOKV outdoors where they probably interact. Similarly, in the United States of America (USA), the number of domestic cat rabies cases is often greatest in the states where raccoon rabies virus (RRV) is endemic [38]. With the continued rabies outbreaks in domestic dogs and their close association with the domestic cats thereof, it would be expected that most rabies cases would be of the canid RABV variant. A similar phenomenon has been reported in the USA where an overwhelming proportion of rabid cats (99%) in the New York State were infected with (RRV) as opposed to a bat rabies variant [39]. In contrast to South Africa, canine rabies has been eliminated in the USA, and sylvatic (wildlife) rabies is a significant challenge, with cats being the most reported animal for rabies among domestic species [40]. The presence of both domestic and wildlife rabies cycles and spillover events into the domestic and wildlife cats reflect a potential public health hazard. The data presented here though, do not support whether the domestic cats or African wild cats are involved in the rabies epidemiological cycles in South Africa or only serve as dead-end hosts as shown in de Lima et al., 2023. In other retrospective studies, it was shown that 94% of the viruses isolated from the domestic cats in KwaZulu Natal were of canid RABV variant, which is the most prevalent variant of RABV in this region of South Africa (6). The RABV sequences determined in this study indicate a continued and persistent rabies epidemiological cycle in domestic dogs and wildlife species, which spillover to other animal species. Massive animal vaccination campaigns globally have created an “environmental barrier” of immune protection and the source of rabies transmission to humans has thus switched from vaccinated to unvaccinated animals, i.e., from dogs to bats and cats. Undeniably, the households or communities with indoor/outdoor cats that are not vaccinated are at an increased risk of rabies exposure. This is despite the fact that vaccination of dogs and cats is compulsory in the Republic of South Africa according to the Animal Diseases Act #35 of 1984. 

It appears that the MOKV is exclusively endemic to Africa, albeit that only 30 sporadic cases have been reported since its discovery more than 50 years ago, with the majority of these cases identified from South Africa [25,36]. The single or sporadic cases identified so far could be attributed to the fact that the maintenance host species of MOKV is still unknown, and these cases may represent cross-species transmission or spillover events into the domestic cats or other animal species. The newly identified MOKV case reported here, provides yet further confirmation that MOKV epidemiological cycles are well established on the African continent and underreported. The male cat in question (467/11) apparently resided in Phophanyane Falls, near Piggs Peak (Eswatini) and was referred for treatment for an undisclosed ailment across the border in Mpumalanga (South Africa). The cat had no vaccination history nor human bite contact, and yet a positive rabies test was confirmed. Therefore, the lack of detection and isolation of rabies-related viruses such as MOKV is testimony to the non-existence of appropriate surveillance tools and limited diagnostic capabilities, such as typing of lyssaviruses using monoclonal antibody panels, and/or genetic sequencing, which are crucial in the differentiation of lyssaviruses, in general. Antigenic and genetic typing of lyssaviruses allows us to distinguish trends of disease dissemination and infer the source of infection, as antigenic rabies variants are associated with different rabies cycles and species of terrestrial carnivores in the region.

The *p*-distance analysis data indicated that the new MOKV isolate from the Eswatini displayed a nucleotide sequence variation of 7.2% and 8.1% on average at a nucleotide level with previous characterized isolates from South Africa and Zimbabwe, respectively. It appears that, overall, all MOKV isolates included for analysis demonstrated a pattern of clustering underpinned by their geographic locality of origin, similar to observations from previous studies [25,36]. The reservoir host of MOKV is still unknown and remains purely speculative, although bat species cannot be ruled out. The cross-species transmission or spillover of MOKV infection into animals such as domestic cats and dogs leads to dead-end infections. Previous studies suggested that the reservoir host species for MOKV might be a carnivore species that interacts with the domestic cats, but there is a little or no evidence to support this notion [29,36]. A recent study found a high seropositivity of 87.80% to MOKV from bushveld gerbils (*Gerbilliscus leucogaster*) rodent, which could suggest a potential rodent reservoir [41], or simply exposure to a pathogen that cross-reacts with MOKV. There is no active surveillance for MOKV on the continent, and subsequently, the epidemiology of this rabies-related virus remains obscure. Lyssaviruses have a strong association with bats, and MOKV and Ikoma virus (IKOV) are the two exceptions among all the other members of the genus [2].

This study was able to contribute some insights on the involvement of domestic and wild cats in the rabies epidemiological cycles in South Africa. Human rabies cases associated with domestic cat exposures in South Africa are rare, and only less than 3% of human rabies cases in the last 36-years period were recorded [30]. Further, the study demonstrated that 37% (n = 3) of human rabies cases were associated with the mongoose RABV variant, whereas the majority, 63% (n = 5) were due to the canid RABV variant [30]. The close association between humans and domestic cats and the lack of protection with the current inactivated vaccines [21,22,36] lend support to the need to undertake more research into the epidemiology and ecology aspects of MOKV, as well as the reservoir host species. Cats have a typical characteristic of forming large colonies of feral cats where intense intra- and interspecific relationships occur, thus increasing the chances of contact with wild animals infected by the *Lyssavirus rabies*, resulting in transmitting infection to other members in the colony [42]. Therefore, eliminating dog rabies would not completely remove the risk of re-introduction of rabies in domestic cats, since the transmission of mongoose RABV variant has been demonstrated here. Since domestic cats have a keen hunting characteristic, they have the potential to transmit the lyssaviruses to domestic animals such as domestic dogs in regions with low rabies vaccination coverage, and due to the proximity to humans, rabies can then be easily transmitted to humans. 

Rabies virus infection and subsequent disease in humans can be prevented through the prompt application of rabies post-exposure prophylaxis (PEP). The management of human contact cases is currently done with strict adherence to the WHO rabies PEP guidelines [43], resulting in close to 100% efficacy [43]. The use of rabies immunoglobulin (RIG) is recommended in all category III exposures, i.e., single or multiple transdermal bites or scratches, contamination of mucous membranes or broken skin with saliva from animal licks, and exposures due to direct contact with bats [44]. Rabies vaccines and RIG have been available for decades; however, these biologicals are based on rabies virus (RABV) [phylogroup I members only]. The diversity of lyssavirus species has more than doubled over the years, with a growing concern for the efficacy of the currently available biologicals against the range of viruses that are antigenically distinct from RABV. In a recent study, two commercial rabies immunoglobulins (RIG) prepared from human, or equine (ERIG) blood plasma were evaluated against a panel of 13 diverse lyssavirus species [43]. The study showed a reduced neutralization for the majority of lyssaviruses compared to rabies virus, particularly for lyssaviruses in phylogroups II and III [43]. Furthermore, neutralization of more diverse lyssaviruses only occurred at very high virus doses, except for Ikoma lyssavirus, which could not be neutralized by the RIG evaluated in this study. Nonetheless, the child who was bitten by the African civet (from which IKOV was recovered) received appropriate wound care and post-exposure rabies vaccination. At the time of the report, the child remained well. The use of RIG is, therefore, a crucial component of rabies post-exposure prophylaxis, but RIG, in its current form, appears not to protect against all lyssaviruses [43]. In addition, higher doses of RIG may be required for neutralization as the genetic distance from vaccine strains increases. Given the limitations of current RIG preparations, alternative passive immunization options should be investigated. Therefore, continuous rabies vaccination of domestic cats remains key, even after the elimination of dog rabies, as the spillover related to a sylvatic rabies cycle will continue to occur.

## Figures and Tables

**Table 1:** Percentage difference (pairwise distances) of the nucleoprotein (N) gene of *Lyssavirus mokola* (MOKV).

MOKV Isolate	14/024	12/604	12/458	U22843	RA133/82	226/08	21846	13270	252/97	12574	12341	700/70	543/95	322/96	229/97	173/06	112/96	071/98	86101RCA	8720SA	86100CAM
14/024																					
12/604	0.002																				
12/458	0.003	0.001																			
U22843	0.072	0.073	0.075																		
RA133/82	0.071	0.072	0.073	0.068																	
226/08	0.037	0.035	0.037	0.069	0.074																
21846	0.063	0.063	0.064	0.057	0.068	0.06															
13270	0.072	0.073	0.075	0	0.068	0.069	0.057														
252/97	0.012	0.011	0.012	0.072	0.068	0.035	0.06	0.072													
12574	0.075	0.076	0.077	0.002	0.068	0.072	0.059	0.002	0.075												
12341	0.072	0.073	0.075	0	0.068	0.069	0.057	0	0.072	0.002											
700/70	0.021	0.02	0.021	0.069	0.072	0.022	0.057	0.069	0.017	0.072	0.069										
543/95	0.033	0.032	0.033	0.067	0.071	0.009	0.059	0.067	0.031	0.069	0.067	0.017									
322/96	0.035	0.034	0.035	0.065	0.071	0.011	0.059	0.065	0.033	0.067	0.065	0.019	0.002								
229/97	0.012	0.011	0.012	0.072	0.068	0.035	0.06	0.072	0	0.075	0.072	0.017	0.031	0.033							
173/06	0.034	0.033	0.034	0.066	0.069	0.01	0.061	0.066	0.03	0.068	0.066	0.018	0.003	0.005	0.03						
112/96	0.034	0.033	0.034	0.068	0.072	0.01	0.061	0.068	0.032	0.071	0.068	0.018	0.001	0.003	0.032	0.004					
071/98	0.009	0.007	0.008	0.068	0.069	0.032	0.058	0.068	0.004	0.071	0.068	0.014	0.029	0.031	0.004	0.03	0.03				
86101RCA	0.079	0.078	0.077	0.068	0.078	0.075	0.072	0.068	0.074	0.071	0.068	0.077	0.073	0.073	0.074	0.074	0.074	0.074			
8720SA	0.021	0.02	0.021	0.069	0.072	0.022	0.057	0.069	0.017	0.072	0.069	0	0.017	0.019	0.017	0.018	0.018	0.014	0.077		
86100CAM	0.071	0.072	0.073	0.068	0	0.074	0.068	0.068	0.068	0.068	0.068	0.072	0.071	0.071	0.068	0.069	0.072	0.069	0.078	0.072	
467/11	0.073	0.071	0.073	0.08	0.075	0.079	0.077	0.08	0.072	0.082	0.08	0.068	0.072	0.072	0.072	0.073	0.071	0.07	0.083	0.068	0.075

## Data Availability

The nucleotide sequence data generated in this study can be found on GenBank.

## Supplementary Materials

The following supporting information can be downloaded at: https://www.mdpi.com/article/10.3390/pathogens12101212/s1, Table S1: Number of viral isolates used in the study; Table S2: Number of *Lyssavirus mokola* included in the analysis.
