# Supplementary material for: Molecular Characterization of Lyssaviruses Originating from Domestic and Wild Cats Provides an Insight on the Diversity of Lyssaviruses and a Risk of Rabies Transmission to Other Susceptible Mammals and Humans in South Africa
Source: Pathogens. 2023 Oct 2;12(10):1212. doi: 10.3390/pathogens12101212 (PMC10609916; doi:10.3390/pathogens12101212)
Supplement: Supplementary file 1 [file pathogens-12-01212-s001.zip › pathogens-2595835-supplementary.pdf]

Table S1: Number of isolates used in the study.

Laboratory number	Year of isolation	Species	Locality	Province	Accession number	Reference
364/96	1996	*Cynictis penicillata*	Uitenhage	Eastern Cape	FJ392379	Van Zyl et al., 2010
228/01	2001	*Ovine*	Kalkdam farm	Free Sate	FJ392376	Van Zyl et al., 2010
155/03	2003	*Galerella sanguine a*	Kroonstad	Free State	FJ392373	Van Zyl et al., 2010
381/06	2006	*Cynictis penicillata*	Hoopstad	Free State	FJ392380	Van Zyl et al., 2010
627/10	2010	*Felis lybica*	Kimberly	Northern Cape	OR192206	This study
708/10	2010	*Felis lybica*	Polokwane	Limpopo	OR192207	This study
871/10	2010	*Felis lybica*	Kroonstad	Free State	OR192208	This study
1006/10	2010	*Felis lybica*	Bethlehem	Free State	OR192209	This study
1082/10	2010	*Felis catus*	Bethlehem	Free State	OR192210	This study
1131/10	2010	*Felis catus*	Skukuza	Mpumalanga	OR192211	This study
68/11	2011	*Felis lybica*	Bethlehem	Free State	OR192212	This study
114/11	2011	*Felis lybica*	Kroonstad	Free State	OR192213	This study
246/11	2011	*Felis catus*	Ficksburg	Free State	OR192214	This study
376/11	2011	*Felis catus*	Bethlehem	Free State	OR192215	This study
467/11	2011	*Felis catus*	Nelspruit	Mpumalanga	OR192216	This study
520/11	2011	*Felis lybica*	Lichtenburg	North-West	OR192217	This study
613/11	2011	*Felis lybica*	Hoopstad	Free State	OR192218	This study
660/11	2011	*Felis lybica*	Bethlehem	Free State	OR192219	This study
689/11	2011	*Felis lybica*	Clocolan	Free State	OR192220	This study
846/11	2011	*Felis lybica*	Kroonstad	Free State	OR192221	This study
457/12	2012	*Felis catus*	Oppaslaagte	North-West	KT892014	Sabeta et al., 2018
555/12	2012	*Canis mesomelas*	Olivenboom	North-West	KT892004	Sabeta et al., 2018
556/12	2012	*Canis familiaris*	Modderkuil	North-West	KT892003	Sabeta et al., 2018
650/12	2012	*Felis catus*	Hennenman	Free State	OR192189	This study
651/12	2012	*Felis catus*	Brandfort	Free State	OR192190	This study
660/12	2012	*Felis catus*	Winburg	Free State	OR192191	This study
718/12	2012	*Felis lybica*	Senekal	Free State	OR192192	This study
784/12	2012	*Felis lybica*	Bloemfontein	Free State	OR192193	This study
797/12	2012	*Felis catus*	Makhado	Limpopo	OR192194	This study

859/12	2012	*Felis catus*	Ermelo	Mpumalanga	OR192195	This study
948/12	2012	*Felis catus*	Welkom	Free State	OR192196	This study
994/12	2012	*Felis catus*	Kimberly	Northern Cape	OR192197	This study
409/13	2013	*Felis catus*	Klerksdorp	North-West	OR192198	This study
432/13	2013	*Felis catus*	De Aar	Northern Cape	OR192199	This study
520/13	2013	*Felis lybica*	Potchefstroom	North-West	OR192200	This study
712/13	2013	*Felis lybica*	Lekwa	Mpumalanga	OR192201	This study
471/14	2014	*Felis lybica*	Wepener	Free State	OR192187	This study
610/14	2014	*Felis catus*	Kroonstad	Free State	OR192171	This study
642/14	2014	*Felis catus*	Frankfort	Free State	OR192172	This study
889/14	2014	*Felis lybica*	Thabazimbi	Limpopo	KT891999	Sabeta et al., 2018
324/15	2015	*Felis catus*	Bethlehem	Free State	OR192184	This study
385/15	2015	*Felis lybica*	Marquard	Free State	OR192185	This study
578/15	2015	*Felis catus*	Potchefstroom	North-West	OR192173	This study
699/15	2015	*Felis lybica*	Lindley	Free State	OR192174	This study
109/16	2016	*Felis catus*	Pretoria	Gauteng	OR192179	This study
230/16	2016	*Felis catus*	Tzaneen	Limpopo	OR192181	This study
279/16	2016	*Felis lybica*	Kroonstad	Free State	OR192182	This study
320/16	2016	*Felis lybica*	Kroonstad	Free State	OR192183	This study
452/16	2016	*Felis lybica*	Zastron	Free State	OR192186	This study
691/16	2016	*Felis lybica*	Lindley	Free State	OR192175	This study
806/16	2016	*Felis catus*	Lanseria	Gauteng	OR192176	This study
830/16	2016	*Felis lybica*	Ladybrand	Free State	OR192177	This study
12/17	2017	**Feline spp.*	Calvinia	Northern Cape	OR192178	This study
541/17	2017	**Feline spp.*	Parys	Free State	OR192188	This study
72/18	2018	**Feline spp.*	Frankfort	Free State	OR192202	This study
156/18	2018	*Felis catus*	iLembe	KwaZulu Natal	OR192180	This study
40/20	2020	*Felis lybica*	Klerksdorp	North-West	OR192203	This study
89/20	2020	*Felis lybica*	Kroonstad	Free State	OR192204	This study
182/20	2020	*Felis catus*	Colesberg	Northern Cape	OR192205	This study

*The name of species is not well written on the submission form.

Table S2: The number of rabies related viruses included in the study for analysis, which include Duvenhage virus, Mokola virus and Lagos bat virus.

Laboratory number	Year of Isolation	Species	Country of Origin	Accession number	Reference
700/70	1970	Domestic cat	Republic of South Africa, KwaZulu Natal	FJ465416	Kgaladi, J., et al., 2013
8720SA	1987	Domestic cat	Republic of South Africa	GU992313	Vandekerkhove, J., et al., 2010
543/95	1995	Domestic cat	Republic of South Africa, Eastern Cape	FJ465415	Kgaladi, J., et al., 2013
112/96	1996	Domestic cat	Republic of South Africa, Eastern Cape	FJ465411	Kgaladi, J., et al., 2013
322/96	1996	Domestic cat	Republic of South Africa, Eastern Cape	FJ465414	Kgaladi, J., et al., 2013
229/97	1997	Domestic cat	Republic of South Africa, KwaZulu Natal	FJ465413	Kgaladi, J., et al., 2013
252/97	1997	Domestic cat	Republic of South Africa, KwaZulu Natal	JN944637	Kgaladi, J., et al., 2013
071/98	1998	Domestic cat	Republic of South Africa, KwaZulu Natal	FJ465410	Kgaladi, J., et al., 2013
173/06	2006	Domestic cat	Republic of South Africa, Eastern Cape	FJ465412	Kgaladi, J., et al., 2013
226/08	2008	Domestic cat	Republic of South Africa, Eastern Cape	KC218934	Kgaladi, J., et al., 2013
12/458	2012	Domestic cat	Republic of South Africa, KwaZulu Natal	KP899610	Kgaladi, J., et al., 2013
12/604	2012	Domestic cat	Republic of South Africa, KwaZulu Natal	KP899611	Kgaladi, J., et al., 2013
14/024	2014	Domestic cat	Republic of South Africa, KwaZulu Natal	KP899612	Kgaladi, J., et al., 2013
12341	1981	Domestic cat	Republic of Zimbabwe, Bulawayo	FJ465417	Kgaladi, J., et al., 2013
12538	1981	Domestic cat	Republic of Zimbabwe	U22843	Kissi, B., et al., 1996
12574	1981	Domestic cat	Republic of Zimbabwe, Bulawayo	FJ465418	Kgaladi, J., et al., 2013
13270	1982	Domestic cat	Republic of Zimbabwe, Bulawayo	KC218932	Kgaladi, J., et al., 2013
21846	1993	Domestic cat	Republic of Zimbabwe, Selous	KC218933	Kgaladi, J., et al., 2013
86101RCA	1986	Rodent	Central African Republic	GU992314	Vandekerkhove, J., et al., 2010
86100CAM	1986	Shrew	Republic of Cameroon	GU992310	Vandekerkhove, J., et al., 2010
RA133/82	1989-1990	Domestic cat	Federal Democratic Republic of Ethiopia	AY333111	Mebatsion, T., et al., 1992
Unknown	1970	Human	Republic of South Africa, Limpopo province	EU623437	Van Eden et al., 2011
Unknown	1981	Unidentified bat species	Republic of South Africa, Limpopo province	EU623438	Van Eden et al., 2011
Unknown	2006	Human	Republic of South Africa, Limpopo province	EU623444	Van Eden et al., 2011
UP1540	2015	*Nycteris thebaica*	Republic of South Africa, Limpopo province	KC866301	McCulloch, S.D. 2015
LBVSA2006	2006	*E. wahlbergi*	Republic of South Africa, KwaZulu Natal	EF547452	Coertse et al., 2020
LBV/UP2250	2013	*E. wahlbergi*	Republic of South Africa, KwaZulu Natal	MH643890	Coertse et al., 2020
LBV/UP7398	2017	*E. wahlbergi*	Republic of South Africa, KwaZulu Natal	MH643891	Coertse et al., 2020

LBV/UP8873	2018	*E. wahlbergi*	Republic of South Africa, KwaZulu Natal	MH643892	Coertse et al., 2020
14/070	2014	*E. wahlbergi*	Republic of South Africa, KwaZulu Natal	KP994623	Coertse et al., 2020
